# Worari Poison and Its Effects

**Published:** 1843-12

**Authors:** 


*Worari Poison and its Effects.*—This virulent substance, manufactured for purposes of offence and defence by the Indians of the interior of Guiana, &c., is now ascertained with tolerable correctness to be essentially the juice of the bark of the worari vine, a gourd bearing plant, the fruit of which is of the size of a large orange. Some other plants or material have been said to be occasionally used in its preparation, as strong Indian pepper, two species of snakes, &c.; but if such be really the case, it is probably with the view, chiefly, of throwing a mystery over the process.

The worari vine grows in a sandy soil. The mode of preparing the poison is as follows:—The inner bark or rind of the root (for it is the root only that is used), is scraped off into some vessel. The worarybally-root undergoes the same process; and is mixed with the worari as is the vine of the courampoey. To these, mixed together and well boiled down with some water, the Indians add some peppers, and further boil the whole mass to a thick syrup.

Dr. Hancock has remarked that if such a thing does exist in nature as a direct sedative, in the strictest sense of the term, he should imagine it to be this extraordinary vegetable extract. Its operation on the animal frame is most mysterious. It extinguishes the vital spark without a pang or a struggle, if prepared without any other substance being added, for the most efficient poison is prepared from the worari vine alone. The sensation and effect it produces are extremely analogous to those which arise from excessive bleeding—the animal under its influence sinking from existence in the most placid swoon.

Sir B. Brodie, in two papers read before the Royal Society in 1811 and 1812, has described the results of several experiments performed with this poison, one of which was the following:—

"Some woorara was inserted into a wound in a young cat. She became affected by it in a few minutes, and lay in a drowsy and half-sensible state, in which she continued at the end of an hour and fifteen minutes, when the application of the poison was repeated. In four minutes after the second application, respiration entirely ceased and the animal appeared to be dead, but the heart was still felt acting about one hundred and forty times in a minute. She was placed in a temperature of 85° Fahr., and the lungs were artificially inflated about forty times in a minute. The heart continued acting regularly. When the artificial respiration had been kept up for forty minutes, the pupils of the eyes were observed to contract and dilate on the increase or diminution of light, saliva had flowed from the mouth, and a small quantity of tears was collected between the eye and the eyelids, but the animal continued perfectly motionless, and insensible. At the end of an hour and forty minutes from the same period there were slight involuntary contractions of the muscles, and every now and then there was an effort to breathe. The involuntary motions continued, and the efforts to breathe became more frequent. At the end of another hour the animal, for the first time, gave some signs of sensibility when roused, and made spontaneous efforts to breathe twenty-two times in a minute. The artificial respiration was discontinued. She lay, as if in a state of profound sleep, for forty minutes, when she suddenly awoke and walked away. On the following day she appeared slightly indisposed; but she gradually recovered, and is at this time still alive and in health."

Sir B. Brodie found that the effect of the poison was the more speedy and certain if there were some, but not much hæmorrhage. It appears that the poison may be taken into the stomach without injury, a considerable quantity having been administered to animals by the mouth without producing any sensible effect.—*Lond. Lancet*, Sept. 1843, from *Pharm. Journ.*, August.

